# Numerical Approach to Spatial Deterministic-Stochastic Models Arising in Cell Biology

**DOI:** 10.1371/journal.pcbi.1005236

**Published:** 2016-12-13

**Authors:** James C. Schaff, Fei Gao, Ye Li, Igor L. Novak, Boris M. Slepchenko

**Affiliations:** Richard D. Berlin Center for Cell Analysis and Modeling, Department of Cell Biology, University of Connecticut Health Center, Farmington, Connecticut, United States of America; National Institutes of Health, UNITED STATES

## Abstract

Hybrid deterministic-stochastic methods provide an efficient alternative to a fully stochastic treatment of models which include components with disparate levels of stochasticity. However, general-purpose hybrid solvers for spatially resolved simulations of reaction-diffusion systems are not widely available. Here we describe fundamentals of a general-purpose spatial hybrid method. The method generates realizations of a spatially inhomogeneous hybrid system by appropriately integrating capabilities of a deterministic partial differential equation solver with a popular particle-based stochastic simulator, Smoldyn. Rigorous validation of the algorithm is detailed, using a simple model of calcium ‘sparks’ as a testbed. The solver is then applied to a deterministic-stochastic model of spontaneous emergence of cell polarity. The approach is general enough to be implemented within biologist-friendly software frameworks such as Virtual Cell.

## Author Summary

Mechanisms of some cellular phenomena involve interactions of molecular systems of which one can be described deterministically, while the other is inherently stochastic. Calcium ‘sparks’ in cardiomyocytes is one such example, in which dynamics of calcium ions, which are usually present in large numbers, can be described deterministically, whereas the channels open and close stochastically. The calcium influx through the channels renders the entire system stochastic, but a fully stochastic treatment accounting for each calcium ion is computationally expensive. Fortunately, such systems can be efficiently solved by hybrid methods in which deterministic and stochastic algorithms are appropriately integrated. Here we describe fundamentals of a general-purpose deterministic-stochastic method for simulating spatially resolved systems. The internal workings of the method are explained and illustrated by applications to very different phenomena such as calcium ‘sparks’, stochastically gated reactions, and spontaneous cell polarization.

## Introduction

It is not uncommon for a cell-biological model to include some components that might be stochastic in nature (small copy numbers, rare events), whereas others, if uncoupled, would behave deterministically (large copy numbers, fast reactions). Through their interaction, fluctuations in a stochastic subsystem may induce significant random perturbations in the ‘deterministic’ one, thus rendering the entire system stochastic. Calcium sparks in cardiomyocytes and other cells [1] is one such example, where calcium released from intracellular stores through calcium channels may, in turn, influence probabilities of opening and closing of those channels. Here, calcium concentration can often be regarded as a ‘deterministic’ component of the system, whereas the dynamics of calcium channels is inherently stochastic. Similarly, stochastic openings of voltage-sensitive ion channels depend on the ‘deterministic’ membrane potential, which, in turn, is affected by stochastic electric currents passing through the channels [2]. Stochasticity in otherwise deterministic cellular subsystems may also be brought about by their coupling to dynamics of cytoskeletal filaments, translation events, and other processes involving macromolecules and small organelles present in small numbers.

Simulating such systems as fully stochastic can be prohibitively slow. Indeed, simulating calcium sparks stochastically with an account of every single calcium ion would be computationally expensive because their number is typically large. But in the limit of large copy numbers, the intrinsic fluctuations due to discreteness of molecules are insignificant, and one can design faster hybrid algorithms, in which deterministic and stochastic approaches are appropriately combined. While these efficient methods are approximate, the larger the copy numbers in the ‘deterministic’ subsystem, the more accurate their outcome.

Numerical approaches to interacting systems with disparate levels of stochasticity are an area of active interdisciplinary research. In the context of cell-biological applications, various hybrid approaches were proposed for ‘well-mixed’ models of biochemical networks with fast and slow components [3, 4]. In these models, a fast component, whose copy numbers are only moderately large, is often modeled as a Wiener stochastic process, rather than deterministically. The corresponding numerical techniques are a combination of methods of solving stochastic ordinary differential equations (SDEs) [5], also termed Langevin equations in the physics literature, and Gillespie-type algorithms [6, 7] that simulate stochastic reaction events in the slow component. Unlike *stochastic* hybrids, the *deterministic*-stochastic models are mathematically defined as piecewise deterministic Markov processes [8, 9], in which the system develops deterministically between consecutive stochastic events. Numerical approaches to such systems are based on a formulation that couples differential equations, which describe continuous variables, with equations that govern probability distributions of the stochastic components. The coupling occurs through ‘deterministic’ rates dependent on discrete stochastic variables and transition probability rates that are functions of continuous variables. Efficient numerical methods for solving deterministic-stochastic models rely on generating Monte Carlo realizations of a hybrid system. For this, a kinetic Monte Carlo algorithm advancing a stochastic subsystem in time must work in conjunction with a deterministic integrator that updates continuous variables by solving the corresponding differential equations.

A variety of algorithms were proposed for spatially uniform, or well-mixed, deterministic-stochastic models. Fixed time step methods, applied to hybrid models of membrane potential [2] and calcium dynamics [10], are conceptually straightforward but incur time-discretization errors in stochastic variables. In adaptive methods, which were first proposed for solving deterministic-stochastic models of biochemical networks [11], the treatment of a stochastic subsystem is essentially free of time-discretization errors. In these algorithms, accurate sampling of stochastic reaction events coupled to continuous variables is achieved by adapting Gillespie’s methods for systems whose transition rates explicitly depend on time. A similar approach was used in a hybrid *stochastic* algorithm for well-mixed systems with fast and slow components [12]. It should be noted that in adaptive methods, special care is required for ensuring synchronous treatment of the ‘deterministic’ and stochastic subsystems. A rigorous convergence analysis of the hybrid adaptive methods was given in [13].

Numerical methods for spatially resolved deterministic-stochastic models are less common. A method described in [14] approximates a stochastic subsystem by a reaction-diffusion master equation [15–18]. In this approximation, a spatial domain is partitioned into subvolumes which are assumed to be well-mixed at any time, and a state of the stochastic subsystem is described in terms of copy numbers per subvolume. The master equation is then solved by an optimized variant of the Next Subvolume method [19]. Designed for models with relatively slow deterministic dynamics, the method of [14] is applicable only if the stochastic subsystems involve sufficiently large copy numbers per subvolume [20, 21].

Stochastic subsystems with relatively low copy numbers can be described in terms of states and spatial locations of individual molecules. The particle-based approach was used to simulate a simple model of assembly of RNA granules in which RNA molecules bind to core complexes [22]. In the model, spatial distributions of RNA molecules were modeled deterministically by partial differential equations. The core complexes and RNA granules were treated stochastically as individual particles interacting with the deterministic subsystem while undergoing random walks. A similar approach was adopted in modeling actin bundles and asters [23, 24], where the stochastic subsystem was comprised of tips of actin filaments while ‘deterministic’ actin monomers were modeled as well-mixed because of their relatively fast diffusion.

States and positions of individual channels were also used to define stochastic subsystems in spatial versions of the deterministic-stochastic models of membrane potential [25] and calcium release from inositol 1,4,5-trisphosphate (InsP_3_)-receptor channels [26–28, 2]. Algorithmically, the methods in these studies combined deterministic descriptions in terms of partial differential equations and the event-driven time stepping schemes [11]. Calcium-induced calcium release in cardiac muscle cells [29] was already mentioned above as a mechanism that naturally lends itself to a hybrid numerical treatment. Playing a key role in ensuring robustness of heart contractions in response to action potentials, it has been studied extensively by various methods [30], including mathematical modeling [31]. The calcium release in cardiomyocytes occurs by way of clustered ryanodine receptor channels (RyR) and, in a healthy heart, takes the form of an avalanche of calcium ‘sparks’, the localized spikes of calcium concentration [32]. Recent advances in experimental technologies have generated renewed interest in detailed predictive computational modeling of calcium dynamics in heart muscle cells for normal and pathological conditions [33, 34]. Similar to calcium release from the (InsP_3_)-receptor channels, the problem entails coupling of a spatial deterministic description of calcium and stochastic kinetics of RyR channels and can be solved efficiently by a hybrid numerical method.

All of the above approaches were largely specific solutions to a specific modeling problem or a restricted domain of problems. In this article, we describe a general-purpose spatial deterministic-stochastic algorithm and discuss techniques used for its validation. The work was motivated by the need of providing tools for simulating spatial hybrid models to a wide range of cell scientists. The method is designed to be applicable to a broad spectrum of models, including those where continuous and discrete variables are defined both in volume and in the encompassing membranes. The current implementation of the method appropriately combines capabilities of one of the Virtual Cell (VCell) [35–39] spatial deterministic solvers and an efficient particle-based simulator called Smoldyn [40, 41]. (Note that Smoldyn has been recently adapted to accommodate a different type of hybrid stochastic models [42], in which the subsystems with disparate levels of stochasticity are segregated in space but can interact in a ‘handshaking’ region [43–46].) The development of the VCell hybrid solver benefited from recent integration of Smoldyn into VCell as a method of solving spatial stochastic models [47]. A distinct feature of our hybrid solver is that the simulations of widespread fluctuations originating from point sources can be carried out in realistic geometries taken from experimental images, as both VCell and Smoldyn provide tools for simulating reaction-diffusion systems in arbitrary geometries [48, 49, 40].

This article is focused on physical underpinnings of the method and its algorithmic details, with special emphasis on rigorous validation of its key elements. Hybrid algorithms, often proposed heuristically, may appear intuitive, but their rigorous analysis and validation constitute a challenging task [12, 25, 7]. This is particularly true in the context of spatially resolved models. Tests against deterministic limits, while necessary, are insufficient because convergence to a correct deterministic limit does not yet guarantee correct behavior in the stochastic regime. Analytical solutions of stochastic models, required for convergence studies in the stochastic regime, are rare, particularly for spatial hybrid systems. In addition to truncation errors due to the time-space discretization, common to deterministic integrators, probability distributions and correlation functions obtained by Monte Carlo techniques include statistical errors due to finite numbers of realizations. Thus, the validation of a spatial hybrid solver entails analysis of multidimensional datasets representing multiple realizations of a hybrid system obtained with varying discretization parameters.

The paper is organized as follows. The algorithm, along with its mathematical fundamentals, is described in Section *Mathematical problem and algorithm* using a simple model of calcium sparks as an example. It is then applied to two very different cell-biological phenomena. The calcium spark model introduced in Section *Mathematical problem and algorithm* is used in Section *Validation of the method* for validation of the method against analytical results and numerical solutions obtained by alternative methods. In Section *Application to a hybrid model of spontaneous cell polarization*, the method is applied to a hybrid model of spontaneous cell polarization; the actual VCell MathModel script for this application is included in S3 Text as an illustration of the software implementation. A summary of results and discussion of possible improvements conclude the paper.

## Results

### The mathematical problem and algorithm

Mathematically, the algorithm is based on a formulation of a deterministic-stochastic system, which is somewhat similar to how Wiener processes are described in terms of Langevin equations. To illustrate the approach and explain the workings of the algorithm, we employ a simple model of calcium sparks, whose ‘deterministic’ subsystem consists of a single variable, the calcium concentration *U*(**r**,*t*), and its stochastic subsystem is comprised of calcium channels, through which calcium flows into the cell from intracellular stores. In muscle cells, calcium channels form small regularly distributed clusters. For simplicity, we will treat the calcium sources as single channels having two states, open and closed. The corresponding discrete stochastic variables are *Ξ*_*i*_(**r**,*t*) ≡ δ(**r** − **r**_*i*_)*ξ*_*i*_(*t*), where the Dirac deltas δ(**r** − **r**_*i*_) define channel locations and the stochastic variables *ξ*_*i*_(*t*) accept two values: 1 (open state) and 0 (closed state). The index *i* enumerates the channels, and **r**,**r**_*i*_ ∈ *Ω*_**cell**_, where *Ω*_**cell**_ denotes the space of a cell.

Dynamics of the continuous variable *U*(**r**,*t*) are affected by the following mechanisms: calcium release through channels, calcium diffusion, and removal of calcium from the cytosol via calcium pumps. The variable is therefore governed by a partial differential equation (PDE) with stochastic source terms,

$$\partial_{t}U=\nabla \cdot (D\nabla U)+J(\sum_{i=1}^{N_{\text{ch}}}\Xi_{i}(r,t))-V_{\text{p}}(U-U_{0}),$$ (1)

where *D* is the calcium diffusion constant, *J* is the calcium flux through an open channel, *N*_ch_ is the total number of channels in the cell, *V*_p_ is the calcium pump rate constant, and *U*_0_ is the steady-state calcium concentration in the absence of open channels. Eq (1) is subject to boundary conditions imposed at the cell membrane. For example, if calcium fluxes at the plasma membrane can be ignored, the corresponding no-flux boundary condition can be written as

$$-D(n\cdot \nabla U){|}_{\partial \Omega_{\text{cell}}}=0,$$ (2)

where **n** is an outward normal to the cell membrane ∂*Ω*_cell_.

Dynamics of the stochastic subsystem are described by a two-component probability distribution function, $\left\{P_{0}^{i}(t),P_{1}^{i}(t)\right\}$, given that in our simple model a channel has only two states. The differential Chapman-Kolmogorov equation that governs Markov processes [5] reduces in this case to

$$\begin{array}{l} P_{0}^{i}(t+dt)=(1-k_{\text{on}}^{i}dt)P_{0}^{i}(t)+k_{\text{off}}^{i}P_{1}^{i}(t)dt\\ P_{1}^{i}(t+dt)=(1-k_{\text{off}}^{i}dt)P_{1}^{i}(t)+k_{\text{on}}^{i}P_{0}^{i}(t)dt \end{array}\;(i=1,2,\ldots ,N_{\text{ch}}),$$ (3)

where $k_{\text{on}}^{i}$ and $k_{\text{off}}^{i}$ are the rate constants for channel openings and closings, respectively. (Because $P_{0}^{i}(t)+P_{1}^{i}(t)\equiv 1$, it is sufficient to solve only for one of the components, say, for $P_{1}^{i}(t)$.) Importantly, parameters $k_{\text{on}}^{i}$ and $k_{\text{off}}^{i}$ may depend on *U*(**r**,*t*); this would couple Eq (3) with Eqs (1 and 2) and also make the equations with different *i*, which otherwise would be independent, indirectly affect each other. Note that because of coupling with *Ξ*_*i*_(**r**,*t*), *U*(**r**,*t*) also becomes a stochastic variable.

Eqs (1–3) fully determine the time-dependent behavior of the deterministic-stochastic system for given initial conditions $\left\{U(r,0);\{P_{1}^{i}(0)\}\right\}$. Their generalization to multivariate (multistate) models is straightforward, yielding descriptions that retain the structure and features of Eqs (1–3). Specifically, a multivariate spatial piecewise-deterministic Markov process is defined in terms of random variables of two types [16, 28], the continuous ‘*U*-type’ and discrete ‘*Ξ*-type’ variables. Using vector notation for sets of these variables, all possible outcomes of the process, {**U**(**r**),**Ξ**(**r**)}, form an infinite-dimensional function space [28]. The only practical approach to solving numerically for a time-dependent probability density functional *p*({**U**(**r**),**Ξ**(**r**)},*t*) is by Monte Carlo simulations of individual realizations of a system based on generation of pseudorandom numbers. The description in the mold of Eqs (1–3) provides an intuitive script for an algorithm of this type. (Alternatively, one can seek a direct numerical solution of a functional equation governing *p*({**U**(**r**),**Ξ**(**r**)},*t*) [5], which, however, quickly runs into memory constraints. Still, this approach can be used for testing purposes, see subsection *Fully coupled systems with finite diffusion*: *validation against direct solutions of Fokker-Planck equation*).

Our spatial hybrid algorithm employs fixed time step integration due to its conceptual and logistical simplicity. The downside is that the stability constraints imposed on the time step, which should be sufficiently small to resolve fast ‘deterministic’ reactions, may result in slow performance. The inefficiency can be partially alleviated by applying an automatic pseudo-steady-state treatment [50].

A key element of a hybrid method is how the numerical treatments of the ‘deterministic’ and stochastic subsystems are merged. In our algorithm, the PDEs are discretized in space using a finite-volume scheme [51], in which a computational domain *Ω* is partitioned into *N*_*ω*_ subvolumes: *Ω* = {*ω*_*j*_}, *j* = 1,…, *N*_*ω*_. The *U*-type variables are discretized respectively as **U**(**r**) → {**U**_*j*_ ≡ **U**(**r**_*j*_)}, where **r**_*j*_ is the center of *ω*_*j*_ and **U**_*j*_ has a meaning of a subvolume average: $U_{j}=|\omega_{j}{|}^{-1}\int_{\omega_{j}}U(r)d^{3}r$, where |*ω*_*j*_| stands for the volume of *ω*_*j*_. Spatial histograms of stochastic variables that use the same subvolumes {*ω*_*j*_} as bins would have the similar meaning. Indeed, let *N*_*Ξ*_ be the number of particles of a given type *Ξ*; then the histogram $\Xi_{j}^{s}=|\omega_{j}{|}^{-1}\int_{\omega_{j}}\sum_{i=1}^{N_{\Xi }}\Xi_{i}(r){|}_{\xi_{i}=s}d^{3}r$ describes the density of particles of the molecular type *Ξ* in state *s* in the vicinity of **r**_*j*_ or, more precisely, the number of particles *Ξ*_*i*_|_*ξ* = *s*_ in *ω*_*j*_ divided by the volume of *ω*_*j*_ (*s* = 1,…, *N*_st_(*Ξ*); here, *N*_st_(*Ξ*) is the number of states of a particle of type *Ξ*). For example, the spatial binning of the stochastic source term of Eq (1) yields $\left|\omega_{j}{|}^{-1}\int_{\omega_{j}}\sum_{i=1}^{N_{\text{ch}}}\Xi_{i}(r){|}_{\xi_{i}=1}d^{3}r=n_{j}/|\omega_{j}\right|$, where *n*_*j*_ is the number of open calcium channels inside *ω*_*j*_. Then, as expected, *Jn*_*j*_/|*ω*_*j*_| is the rate of change of calcium concentration due to the influx through open channels located in the vicinity of **r**_*j*_. As a result, both the deterministic and stochastic rates can now be expressed in terms of sets $\left\{U_{j},\Xi_{j}^{s}\right\}$ with components defined for the same spatial grid, which makes advancing the hybrid system in time conceptually straightforward.

A realization of a piecewise deterministic Markov process at time *t* + *Δt* is generated on the basis of a known state at time *t* as follows. For sufficiently small time steps *Δt*, such that the sum of the *O*(*Δt*) terms in the expansion of the total transition probability for a particle is less than 1, a particle may undergo at most one stochastic transition per *Δt* from its current state to a new one. (For the example described by Eq (3), this requirement yields a condition $\Delta t<<1/\max(k_{\text{on}}^{i},k_{\text{off}}^{i})$). Thus, without loss of generality, occurrences of the stochastic transitions can be assigned to the end points of the interval *Δt*. Therefore *during* the interval, the variables **Ξ**(**r**,*t*) remain unchanged and, upon the binning described above, the equations for **U**(**r**,*t*) become regular deterministic PDEs (see, e.g., Eq (1) of the simple calcium sparks model). The updated values **U**(**r**,*t* + *Δt*) are then found by integrating the PDEs over *Δt* with the corresponding boundary conditions (exemplified by Eq (2)). In our method, this is done by employing a fixed time step PDE solver of VCell.

The update of variables **Ξ**(**r**,*t*) is carried out by employing Smoldyn, a particle-based fixed time step Monte Carlo package [40, 41]. Using again the simple calcium spark model as an example, the transitions of a channel between open and closed states can be interpreted as ‘unimolecular’ reactions, which are simulated by Smoldyn through acceptance-rejection sampling. First, those of the rate parameters $k_{\text{on}}^{i}$ and $k_{\text{off}}^{i}$ in Eq (3) that depend on *U*(**r**,*t*) are updated accordingly. Next, given known states of the channels *ξ*_*i*_(*t*) at time *t*, the probability of a transition to occur by the end of the time interval is computed. If, for example *ξ*_*i*_(*t*) = 0, i.e. the *i*th channel is in a closed state at time *t*, then $P_{0}^{i}(t)=1$ and $P_{1}^{i}(t)=0$. As a result, the first of Eq (3) becomes $dP_{0}^{i}/dt=-k_{\text{on}}^{i}P_{0}^{i}$, and because the rate constants stay fixed during the time interval, $P_{0}^{i}(t+\Delta t)=\exp(-k_{\text{on}}^{i}\Delta t)$ and $P_{1}^{i}(t+\Delta t)=1-\exp(-k_{\text{on}}^{i}\Delta t)$. Finally, a random number *r* is generated and compared with $P_{1}^{i}(t+\Delta t)$. If $r<P_{1}^{i}(t+\Delta t)$, the transition to the open state with *ξ*_*i*_(*t* + *Δt*) = 1 is accepted, otherwise it is rejected. The similar logic applies to channels that are open at time *t*.

Note that bimolecular reactions, in which one of the participants is described by a *U*-type variable and the other is represented by a *Ξ*-variable, can be approximated in deterministic-stochastic models as unimolecular. Indeed, the copy numbers described by variables of *U*-type are assumed to be deterministically large even within *ω*_*j*_, so the changes due to binding to, or unbinding from, discrete particles can be ignored. In other words, the molecules described in terms of concentrations could be treated as ‘catalysts’ in this type of interactions.

In summary, the algorithm includes the following steps:

*Initializing the system*:

- Use initial conditions of the problem to initialize variables **U**(**r**,0) and **Ξ**(**r**,0). If the initial condition for *Ξ*-type variables are given by their probability distribution functions *P*(**Ξ**(**r**), 0), then sample *P*(**Ξ**(**r**), 0). The current version of the solver supports sampling of a uniform spatial distribution of particles.
- Use **U**(**r**,0) to initialize the transition rate parameters for the *Ξ*-type variables.
- Compute initial binned densities for *Ξ*-type variables.
  *Advancing the system in time*:
- Determine **U**(**r**,*t* + *Δt*) by integrating the PDEs using **U**(**r**,*t*) and the spatial binning of the *Ξ*-type variables computed in steps (iii) and (vii) as initial conditions. In the current version of the algorithm, this is done by calling the VCell semi-implicit PDE solver.
- Find **Ξ**(**r**,*t* + *Δt*), using **Ξ**(**r**,*t*) as initial conditions. Currently, Monte Carlo routines of Smoldyn are utilized to implement this step.
- Use **U**(**r**,*t* + *Δt*) to update the transition rate parameters for *Ξ*-type variables;
- Use **Ξ**(**r**,*t* + *Δt*) to update the binned densities for *Ξ*-type variables.

### Validation of the method

Accuracy of our spatial deterministic-stochastic solver is affected by truncation errors, arising from discretization of space and time, and statistical errors due to finite numbers of Monte Carlo realizations. The algorithm was validated against analytical results and through comparison with alternative methods. The calcium spark model introduced in the previous section was used as a testbed for the tests described below.

#### Convergence of solutions of a hybrid system with separable variables

If transition parameters for *Ξ*-variables, such as $k_{\text{on}}^{i}$ and $k_{\text{off}}^{i}$ in Eq (3), are independent of *U*-variables, the **Ξ**–subsystem is separable and can be solved independently. For this case, the probabilities of the channel states in the calcium spark model, $\left\{P_{0}^{i}(t),P_{1}^{i}(t)\right\}$, and the expectation values of the spatial average of *U*(**r**,*t*), ${\bar{U}}_{\text{exact}}(t)=E[|\Omega_{\text{cell}}{|}^{-1}\int_{\Omega_{\text{cell}}}U(r,t)dr]$, can be determined analytically by integrating Eqs (1–3), see S1 Text. This allows us to compute solution errors and analyze convergence of hybrid solutions.

**Fig 1:**
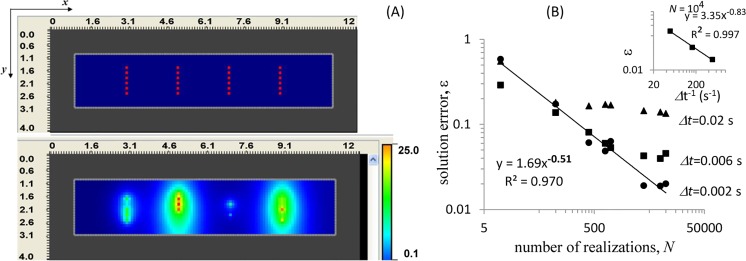
Test with a separable stochastic subsystem. (A) Channel arrangement (upper panel) and a snapshot of simulation results for *U*(**r**,*t*) at *t* = 1 s (lower panel). (B) The multi-trial mean, $<|\Omega_{\text{cell}}{|}^{-1}\int_{\Omega_{\text{cell}}}U(r,t)dr>$, converges to an exact expectation value with the increasing number of trials and decreasing time step *Δt* (data points for *Δt* = 0.02 s, 0.006 s, and 0.002 s are shown as triangles, squares, and circles, respectively). Inset: convergence with respect to *Δt* (shown for *N* = 10^4^).

In this test, we used the hybrid method to solve Eqs (1–3) in a simple quasi-2D geometry *Ω*_cell_ = [0,10.1]×[0,2.1]×[0,0.5] μm^3^ with the arrangement of channels shown in Fig 1A and the following parameter set: *J* = 10 μM·μm^3^/s, *U*_0_ = 0.1 μM, *k*_on_ = 1 s^-1^, *k*_off_ = 5 s^-1^, *V*_p_ = 1 s^-1^, *D* = 1 μm^2^/s, and *N*_ch_ = 24. Simulations, initialized at *ξ*_*i*_(0) = 0 (for all *i* = 1,….*N*_ch_) and *U*(**r**,0) = *U*_0_, were run to *T* = 5 s, at which the system began approaching a steady state.

The solution errors defined as $\varepsilon =\underset{0\leq t\leq T}{\max}|{\bar{U}}_{\text{exact}}-<|\Omega_{\text{cell}}{|}^{-1}\int_{\Omega_{\text{cell}}}U(r,t)dr]{>}_{N}|$, where the angular brackets <…>_*N*_ denote averaging over *N* realizations, were computed for solutions *U*(**r**,*t*) obtained with varying mesh sizes, time steps *Δt* and *N*. Fig 1B demonstrates *ε* as a function of *N* and *Δt* (inset) for solutions computed with the spatial resolution *Δx* = *Δy* = 0.1 μm, *Δz* = 0.5 μm, such that the truncation error is mainly due to discretization of time. For *Δt* = 2 ms, the power fit yields an exponent close to $-\frac{1}{2}$, indicating that for small *Δt*, the solution error, as expected, is largely determined by its statistical component. Overall, the data indicate convergence of $<\bar{U}(t){>}_{N}$ to ${\bar{U}}_{\text{exact}}$, but the validation is limited to cases with separable stochastic subsystems.

#### Fully coupled systems in the limit of fast diffusion: validation against different solvers

One way to validate a hybrid solver for conditions in which the variables are inseparable is to use a fully coupled calcium spark model in the limit of large *D*. Because the system is well-mixed in this limit, a reference solution can be obtained by solving the corresponding fully stochastic system using a non-spatial stochastic simulator.

In the tests, the full coupling was achieved by replacing *k*_on_ with *k*_on_*U*(**r**,*t*)/*U*_0_, and the VCell hybrid solver was run with *k*_on_ = 0.1 s^-1^, *D* = 1000 μm^2^/s and *Δt* = 0.2 ms. All other parameters, as well as the initial conditions, geometry, and mesh, were the same as in the previous subsection. The reference solution was obtained by solving the corresponding well-mixed problem with a VCell nonspatial stochastic solver that implements a ‘next reaction’ algorithm proposed by Gibson and Bruck [7]. This algorithm is an adaptive event-driven method free of time-discretization error (see *Introduction*).

**Fig 2:**
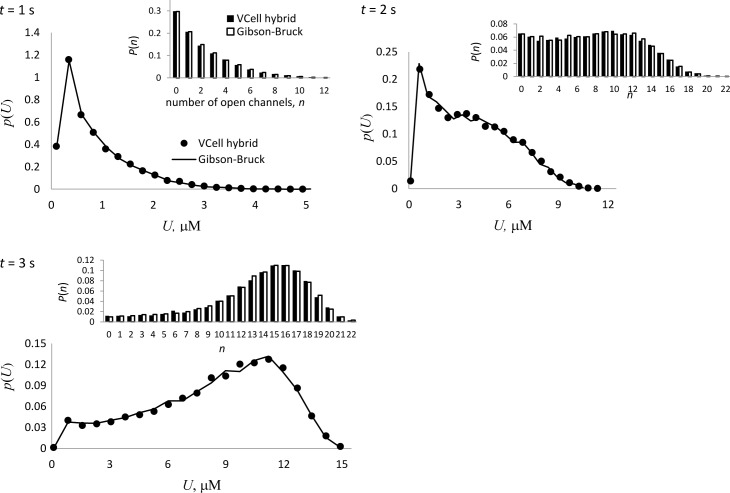
Time-dependent solutions of a fully coupled problem with large *D*. Results from VCell hybrid (dots) are validated against a reference solution of the corresponding well-mixed system obtained by Gibson-Bruck nonspatial solver (solid line). Probability density functions of *U* and probability distributions of the numbers of open channels in insets (black columns for VCell hybrid, white columns for Gibson-Bruck) are based on 10,000 realizations by each solver and shown for three time points, *t* = 1 s, 2 s, 3 s.

**Fig 3:**
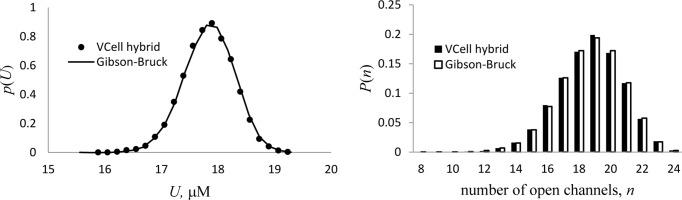
Steady-state solution of a fully coupled system in the limit of large *D*. As in Fig 2, the near steady-state solution of a fully coupled system was obtained by VCell hybrid for the fast-diffusion limit and validated against Gibson-Bruck stochastic nonspatial solver. Results from each solver are based on 20,000 realizations and shown for *t* = 30 s, the time long enough for the system to become sufficiently close to its steady state.

Time-dependent solutions obtained by the two solvers are illustrated in Fig 2. The results, shown for three time points, are based on 10,000 realizations. Because of the large *D*, the realizations of *U*(**r**,*t*) obtained by the VCell hybrid solver were nearly uniform in space for any of the presented times. Still, for comparison with the nonspatial solver, they were averaged over *Ω*_cell_, and the symbol *U*, used in Figs 2 and 3 and below, denotes spatial averages (as well as calcium concentrations in the well-mixed problem). The marginal probability density function *p*(*U*,*t*), defined as $p(U,t)=\sum_{\left\{\xi_{i}\right\}}p(U,\{\xi_{i}\},t)$, where *p*(*U*,{*ξ*_*i*_},*t*) is the joint probability distribution, was approximated by computing a normalized 20-bin histogram over the range of *U* and dividing the frequencies by the lengths of the intervals. Insets of Fig 2 illustrate the probabilities of the number of open channels, which are defined as $P(n,t)=\int_{U}p(U,\{\xi_{i}\},t){|}_{\sum_{i}(\xi_{i})=n}dU$; they were determined by computing the corresponding histograms. Fig 2 demonstrates that the time-dependent solution obtained by the VCell hybrid solver for a fully coupled system in the limit of fast diffusion is consistent with the results obtained by an adaptive nonspatial solver for the corresponding well-mixed model. The differences between the two solutions at *t* = 1 s, 2 s, 3 s computed in L^2^-norm for *p*(*U*,*t*) (first number) and *P*(*n*,*t*) (second number) are as follows: (0.0126, 0.0018), (0.0077, 0.0024), and (0.0043, 0.0026).

Similarly, Fig 3 demonstrates good agreement between the steady state distributions obtained by the spatial hybrid solver for the case of fast diffusion and the corresponding solution of a well-mixed system by the Gibson-Bruck method; the corresponding differences in L^2^-norm are 0.0127 and 0.0017 for *p*(*U*,*t*) and *P*(*n*,*t*), respectively, or ≈1% of the corresponding maximum values. The results in Fig 3 are shown for *t* = 30 s, which is sufficient for accurately approximating *p*(*U*,∞) and *P*(*n*,∞) by *p*(*U*,*t*) and *P*(*n*,*t*) (the solution becomes stationary at *t* ≈ 10 s).

Alternatively, reference solutions of coupled hybrid models in the limit *D* → ∞ can be obtained by solving directly the corresponding Fokker-Planck equations [5]. Unlike the stochastic approach of the hybrid method, direct solution of a Fokker-Planck equation does not involve Monte Carlo techniques and therefore is free of statistical error. While the direct approach is not practical for solving realistic spatial models because of the excessively large dimensionality of a discretized domain, it can be used for testing purposes, for appropriately downsized test problems. Consider for simplicity a system with a single channel placed at the origin. Averaging Eq (1) over *Ω*_cell_ with the account of Eq (2) then yields ∂_*t*_*U* = *Jξ*/|*Ω*_cell_| − *V*_p_(*U* − *U*_0_) with $U=|\Omega_{\text{cell}}{|}^{-1}\int_{\Omega_{\text{cell}}}U(r)dr$. The continuous and discrete variables are again coupled by replacing *k*_on_ with *k*_on_*U*/*U*_0_. Upon nondimensionalization: $\rho =\frac{U-U_{0}}{U_{0}}$, *τ* = *tV*_p_, $\alpha =\frac{k_{\text{off}}}{V_{\text{p}}}$, $\beta =\frac{k_{\text{on}}}{k_{\text{off}}}$, $a=\frac{J}{U_{0}V_{\text{p}}|\Omega_{\text{cell}}|}$, the equation for *U* reduces to $\frac{d\rho }{d\tau }=a\xi -\rho$, while *αβ*(*ρ* + 1) and *α* become the respective dimensionless versions of the ‘on-’ and ‘off-’ parameters for the transition probability rates in Eq (3).

A corresponding joint probability density function *p*(*ρ*,*ξ*,*τ*), defined in this case in a one-dimensional domain {*ρ*,*ξ*} with a two-valued discrete variable *ξ*, has two components: *p*_0_(*ρ*,*τ*) = *p*(*ρ*,*ξ* = 0,*τ*) and *p*_1_(*ρ*,*τ*) = *p*(*ρ*,*ξ* = 1,*τ*). The governing Chapman-Kolmogorov equation then reduces to

$$\begin{array}{l} \partial_{\tau }p_{0}(\rho ,\tau )=-\partial_{\rho }[-\rho p_{0}(\rho ,\tau )]+R\\ \partial_{\tau }p_{1}(\rho ,\tau )=-\partial_{\rho }[(a-\rho )p_{1}(\rho ,\tau )]-R \end{array},$$ (4)

where *R* = *α*(*p*_1_(*ρ*,*τ*) − *β*(*ρ* + 1)*p*_0_(*ρ*,*τ*)). Eq (4) are to be solved with the initial conditions *p*_0_(*ρ*,0) = δ(*ρ*), *p*_1_(*ρ*,0) = 0 and the boundary conditions ∂_*ρ*_*p*_0_|_*ρ* = 0_ = ∂_*ρ*_*p*_1_|_*ρ* = 0_ = 0. Thus, in the limit of fast diffusion, the Fokker-Planck formulation is equivalent to a set of hyperbolic equations (Eq (4)).

**Fig 4:**
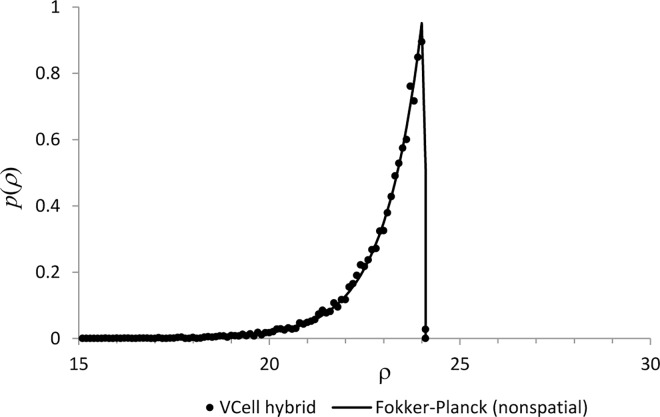
Spatial hybrid vs. Fokker-Planck formulation in the limit of large *D*. Probability density function of dimensionless continuous variable *ρ* is shown for *t* = 30 s, sufficient for accurate approximation of the system’s steady state. Results from VCell hybrid (dots) are based on 10,000 realizations, and Fokker-Planck equations (Eq (4)) were solved by VCell fully implicit advection-diffusion solver with mesh size *Δρ*/*ρ*_max_ = 0.004 (solid curve). The L^2^-norm of the difference between the two solutions is 0.0137.

In the test, the single-channel hybrid system was solved by the VCell hybrid solver in 3D geometry, *Ω*_cell_ = [−0.5,0.5]×[−0.5,0.5]×[−0.5,0.5], for *α* = *β* = 1, *a* = 24. The dimensionless diffusion constant, *d* = *D*/(*V*_p_*l*^2^) with *l* = 1 μm, was set at 10^4^, and the simulations were run with the time step *Δτ* = 0.002 and the mesh sizes *Δx* = *Δy* = *Δz* = 0.1. The results for the probability density function *p*(*ρ*,*τ*) = *p*_0_(*ρ*,*τ*) + *p*_1_(*ρ*,*τ*) are shown in Fig 4 for time *τ* = 30, sufficiently long to accurately approximate the steady-state distribution *p*(*ρ*,∞) with *p*(*ρ*,*τ*). The hybrid solution based on 10,000 realizations (dots) agrees well with the direct solution of Eq (4) obtained by a VCell fully-implicit PDE solver [39] with the mesh size *Δρ* = 0.1 (solid curve).

#### Fully coupled systems with finite diffusion: validation against direct solutions of Fokker-Planck equations

The idea of testing the hybrid solver against a fully stochastic simulator, which worked in the limit of fast diffusion (see previous subsection), turned out to be not particularly practical for testing spatially heterogeneous solutions of hybrid models. As discussed in *Introduction*, treating such models by a fully stochastic spatial solver is computationally expensive, because the copy numbers of ‘deterministic’ species must be large not only in total but locally as well. Also, the probability rates originating from the ‘hybrid’ terms in stochastic subsystems depend on large local copy numbers describing ‘deterministic’ variables and therefore are fast, thus necessitating a small integration time step. As a result, obtaining an accurate solution that entails multiple runs of a fully stochastic solver becomes prohibitively slow.

It is still possible to achieve fairly large local copy numbers for single-channel calcium spark models with low-dimensional geometries. We used Smoldyn to obtain fully stochastic solutions of a quasi-1D coupled model for different total copy numbers of calcium ions and compared them to the corresponding solution obtained by the hybrid solver. As expected, the probability distributions obtained with larger total numbers of particles were closer to the hybrid solution, but suppressing the errors due to the finiteness of the copy numbers describing the ‘deterministic’ variable and due to the finiteness of the number of Monte Carlo trials proved to be challenging because of the constraints described above.

For more accurate comparison, we used direct numerical solution of the Fokker-Planck equation, which, as mentioned earlier, is free of statistical error. In the case of finite *D*, this approach has its own limitations due to an exponential increase of the domain size with the number of spatial degrees of freedom. We therefore again employed the quasi-1D single-channel test problem and solved it on coarse meshes. For finite *D*, the problem is formulated in terms of the probability density functionals, *p*_0_(*ρ*(*x*),*τ*) and *p*_1_(*ρ*(*x*),*τ*). Here and below *x* and *d* are the dimensionless spatial coordinate and diffusion coefficient. The corresponding generalization of Eq (4) yields functional Fokker-Planck equations that include diffusion-related terms [5]:

$$\begin{array}{l} \partial_{\tau }p_{0}(\rho (x),\tau )=R-\int dx\frac{\delta }{\delta \rho (x)}[(d\cdot \hat{L}\rho (x)-\rho (x))p_{0}(\rho (x),\tau )]\\ \partial_{\tau }p_{1}(\rho (x),\tau )=-R-\int dx\frac{\delta }{\delta \rho (x)}[(d\cdot \hat{L}\rho (x)+(a\delta (x)-\rho (x))p_{1}(\rho (x),\tau )] \end{array}.$$ (5)

In Eq (5), *x* ∈ [0,*x*_max_] and for every *x*, *ρ*(*x*) ∈ [0,*ρ*_max_(*x*)], where *ρ*_max_(*x*) define the ranges of possible values of *ρ*(*x*); $\frac{\delta }{\delta \rho (x)}$ is the operator of functional (variational) differentiation, $\hat{L}$ is the diffusion operator, which in the continuous limit can be symbolically written as $\hat{L}=\partial_{xx}^{2}$, and δ(*x*) is the Dirac delta-function. The term describing transitions between the states of the channel, which was placed at the origin as in the fast diffusion test, is *R* = *α*(*p*_1_ − *β*(*ρ*(0) + 1)*p*_0_). Note that because the spatial coordinate *x* has the dimension of length, the units of the normalized diffusion constant, denoted as *D* in Eq (5) and below, are those of length squared. The problem is solved with the initial conditions, *p*_0_(*ρ*(*x*),0) = δ(*ρ*(*x*)), *p*_1_(*ρ*(*x*),0) = 0 and with the zero-flux boundary conditions on all boundaries for *ρ* and *x*. Discretization of Eq (5) and solution of the discretized equations are described in S2 Text.

**Fig 5:**
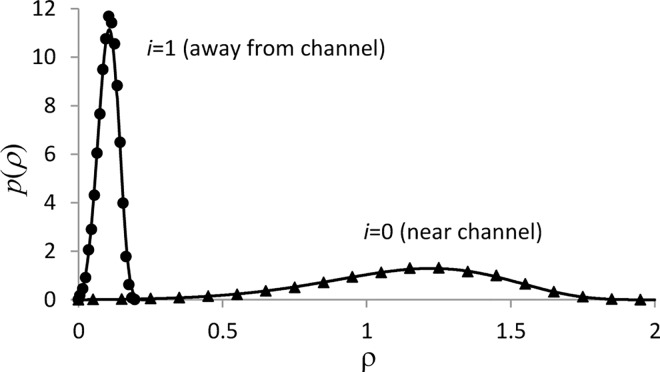
Validation of spatially inhomogeneous solutions (finite *D*), *i*_max_ = 2. Solutions for probability density function *p*(*ρ*) are shown for *τ* = 1. Results from VCell hybrid (triangles for *i* = 0 and circles for *i* = 1) are based on 12,500 realizations. Solutions of the corresponding functional Fokker-Planck equation (solid curves) were obtained with *Δρ* = 1.25e-3. The L^2^-norms of the differences of the two solutions are ≈ 1.9% (*i* = 0) and 3.3% (*i* = 1) of the respective maximum values.

The direct numerical solutions of Eq (5) were compared with the hybrid solutions obtained for the same spatial grid. The hybrid formulation in this case includes the following equation,

$$\partial_{\tau }\rho (x,\tau )=d\cdot \partial_{xx}^{2}\rho (x,\tau )+a\delta (x)\xi (\tau )-\rho (x,\tau ),$$

where *ξ*(*τ*) is the Poisson stochastic process with the ‘on-’ and ‘off-’ rate constants *αβ*(*ρ*(0) + 1) and *α*, respectively. Fig 5 illustrates comparison of the solution of the functional Fokker-Planck equation on the two-dimensional grid, *i*_max_ = 2 (*x*_max_ = 4, *Δx* = 2), and the corresponding solution by the VCell hybrid solver, which were obtained for *α* = 10, *β* = 1, a=203, and *d* = 1. The results are shown for the probability density function *p*(*ρ*(*x*_*i*_),*τ*) = *p*_0_(*ρ*(*x*_*i*_),*τ*) + *p*_1_(*ρ*(*x*_*i*_),*τ*) at *τ* = 1. Integration with the spatial hybrid solver was performed with the time step *Δτ* = 1e-4, and the results are based on 12,500 realizations. The values of *p*(*ρ*(*x*_*i*_),*τ*) (dots in Fig 5) were computed by building 20-bin histograms in [0,*ρ*_max_(*i*)] and dividing the frequencies by *Δρ*. The results obtained by the two very different methods are in overall good agreement. Note that the discrepancies of ≈5% near the maximum of *p*(*ρ*(*x*_1_),*τ*) indicate that the interval [0,*ρ*_max_(1)] is under-resolved, but *Δρ* could not be made significantly smaller due to memory constraints.

**Fig 6:**
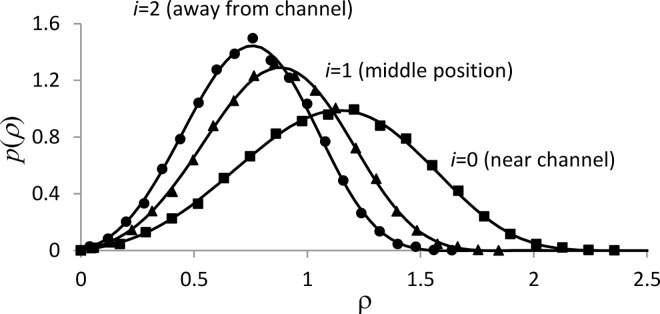
Validation of spatially inhomogeneous solutions (finite diffusion), *i*_max_ = 3. Solutions for probability density function *p*(*ρ*) are shown for *τ* = 1. Results from VCell hybrid for positions near the channel (*i* = 0), squares), away from the channel (*i* = 2, circles) and in between (*i* = 1, triangles) are based on 12,500 realizations. The curves are extrapolations to *Δρ* = 0 of numerical solutions of the corresponding Fokker-Planck equation, computed with *Δρ_s_* ∝ (1.5)*^-s^* 0, *s* = 0,1,…,5. The L^2^-norms of the differences of the two solutions are ≈ 1.3% (*i* = 0 and *i* = 1) and 1.5% (*i* = 2) of the respective maximum values.

These limitations are more restrictive for three-dimensional grids, so the reference data for *i*_max_ = 3 were obtained by extrapolating to *Δρ* = 0 a series of solutions computed with different *Δρ*, an approach that has been applied in a different context in [52] (see S2 Text for details). Fig 6 demonstrates comparison of the extrapolated curves with the solution obtained by the VCell hybrid solver with *i*_max_ = 3 (*x*_max_ = 6, *Δx* = 2) for *α* = 20, *β* = 0.5, *a* = 20, *d* = 50. The hybrid solution was based on 12,500 simulations obtained with *Δτ* = 2e-5. As in the previous test, the values of *p*(*ρ*(*x*_*i*_),*τ*) were computed using the 20-bin histograms calculated for each [0,*ρ*_max_(*i*)].

The good agreement of the solutions obtained for the same spatial grids by the entirely different methods validates the hybrid solver for conditions of slow diffusion and full coupling of the stochastic and ‘deterministic’ components.

#### Other capabilities and limitations of the method

The tests described in the previous subsections verify key elements of the spatial hybrid method exemplified by a simple model of calcium sparks of Section *Mathematical problem and algorithm*. Here we briefly discuss other functionality of the VCell hybrid solver not included in Eqs (1–3) and limitations of the current version of the method.

In the test examples, positions of the channels were fixed but in general, particles constituting a stochastic subsystem can diffuse and/or drift (see Section *Application to a hybrid model of spontaneous cell polarization*). Thus, vectors **r**_*i*_ describing their positions could be continuous functions of time governed by a stochastic process. Also not included in the tests is the binding of discrete particles to one another (the channels in the test problems undergo first-order (unimolecular) reactions). The hybrid solver supports these additional capabilities via Smoldyn [40], which approximates particles as points and implements their diffusion in space by sampling the exact diffusion propagator, which in and of itself does not incur additional numerical errors for any *Δt*. In a hybrid setting, however, diffusion of particles may amplify truncation errors due to discretization of time and space (see *Convergence of solutions of a hybrid system with separable variables*). Indeed, an interaction of a diffusing particle with ‘deterministic’ species can be equivalently described as the interaction between a fixed particle and the ‘deterministic’ species with increased diffusivities. Therefore, to maintain comparable accuracy of computations on a similar spatial grid, simulations of a hybrid model with diffusing particles should be run with appropriately decreased *Δt*.

The bimolecular reactions are simulated by Smoldyn as diffusion-limited, i.e. two particles positioned within a binding distance connect instantaneously. By default, the binding distance is set automatically on the basis of a given *Δt*, diffusion coefficients of the binding partners, and a desired macroscopic rate constant. Whereas with *Δt*→0, the method converges to solutions of reaction-diffusion systems with diffusion-limited reactions, the numerical error due to finite *Δt* results in radial pair distributions that would have been produced if the intrinsic binding occurred with a finite rate [40]. In our hybrid algorithm, these limitations of Smoldyn apply to bimolecular reactions within a stochastic subsystem.

We now consider limitations in treating bimolecular reactions where a continuously described molecule binds to a discrete particle. Because the hybrid approach assumes that the molecules of the ‘deterministic’ subsystem are expressed in large copy numbers, one can ignore sequestration of a continuous variable in such reactions, i.e. approximate a continuously described species as a catalyst (see discussion in section *Mathematical problem and algorithm*). In some applications, however, possible variations of concentrations as a result of such binding may need to be taken into account. For these problems, the hybrid method yields accurate results if diffusion is sufficiently fast, i.e. the binding is dominated by the intrinsic binding and the resulting depletion is small. Note that in the current implementation of our method, the treatment of the binding partners belonging to the different subsystems is slightly asynchronous, see steps (iv) and (v) of the algorithm described in section *Mathematical problem and algorithm*. This causes deviations from local mass conservation, which are usually small but may exacerbate and even cause numerical instability if *Δt* is insufficiently small.

To test our method in the opposite limit of diffusion-influenced reactions that may result in significant depletion of the continuous component in the immediate vicinity of a particle, we applied it to a model of stochastically gated reactions, for which accurate numerical results and analytical asymptotics have been obtained in [53]. In this model, macromolecule *M*, expressed in relatively low numbers, switches stochastically between inert and reactive configurations with the rate constants of activation and inactivation *a* and *b*. When in the reactive configuration, the macromolecule can bind ligand *L* to form complex *C* (see [53] and references therein). The binding is modeled as diffusion-influenced with the reaction rate described as

$$4\pi Dr_{c}^{2}\partial_{r}\rho_{M_{\text{active}}L}(r,t){|}_{r=r_{c}}=\kappa_{\text{f}}\rho_{M_{\text{active}}L}(r_{c},t)-\kappa_{\text{r}}[C],$$

where $\rho_{M_{\text{active}}L}(r,t)$ is the pair distribution function of the ligand and reactive macromolecule; the macromolecule is modeled as a sphere with radius *r*_*c*_; *D* is the sum of diffusion coefficients of *M* and *L*, and *κ*_f_, *κ*_r_ are the forward (binding) and reverse (unbinding) rate constants, respectively. Of interest is the effect of slow diffusion on the relaxation function of reversible binding of the gated receptor, $\frac{C_{\text{eq}}-C(t)}{C_{\text{eq}}-C(0)}$, where *C*(*t*) is the total number of complexes in the space *Ω* occupied by the system at time *t* and *C*_eq_ = *C*(∞). Specifically, it is predicted that the relaxation function has a power-law tail ∝ *t*^−3/2^ at *t* >> *τ*_D_, where $\tau_{\text{D}}=r_{\text{c}}^{2}/D$ [53].

In a hybrid version of the model, the ligands are assumed to be expressed in large copy numbers and are described deterministically by their concentration *L*(**r**,*t*), whereas the macromolecules are represented by discrete variables ${Μ}_{i}(r,t)=\delta (r-r_{i}){\left(\mu_{i}^{\left(1\right)}(t),\mu_{i}^{\left(2\right)}(t),\mu_{i}^{\left(3\right)}(t)\right)}^{T}$ with **r**,**r**_*i*_ ∈ *Ω*, *i* = 1,…,*N*, and *N* is the total number of macromolecules in the system. Each of the stochastic components $\mu_{i}^{\left(\alpha \right)}(t)$ that describe respectively the inert, active, and complex states, assumes values 0 or 1 in such a way that $\sum_{\alpha }\mu_{i}^{\left(\alpha \right)}=1$. We assume that the macromolecules are immobile in all forms and randomly distributed throughout *Ω*.

In the corresponding ‘Langevin-like’ formulation, *L*(**r**,*t*) is governed by the equation,

$$\partial_{t}L(r,t)=D\nabla^{2}L(r,t)-\kappa_{\text{f}}L(r,t)\sum_{i=1}^{N}{Μ}_{i}(r,t){|}_{\mu_{i}^{\left(2\right)}=1}+\kappa_{\text{r}}\sum_{i=1}^{N}M_{i}(r,t){|}_{\mu_{i}^{\left(3\right)}=1},$$ (6)

which is subjected to a Dirichlet boundary condition, *L*(**r**,*t*)|_∂*Ω*_ = *L*_0_ = 1 μM ≈ 602 μm^-3^, and realizations of $\mu_{i}^{\left(\alpha \right)}(t)$ are governed by Poisson processes with the following transition probabilities,

$$\begin{array}{l} p(\alpha =2,t+dt)|\alpha =1,t)=adt,\;p(\alpha =1,t+dt)|\alpha =1,t)=1-adt\\ p(\alpha =1,t+dt)|\alpha =2,t)=bdt,\;p(\alpha =3,t+dt)|\alpha =2,t)=\kappa_{\text{f}}L(r,t)dt\\ p(\alpha =2,t+dt)|\alpha =2,t)=1-bdt-\kappa_{\text{f}}L(r,t)dt\\ p(\alpha =2,t+dt)|\alpha =3,t)=\kappa_{\text{r}}dt,\;p(\alpha =3,t+dt)|\alpha =3,t)=1-\kappa_{\text{r}}dt \end{array},$$ (7)

where the states are specified by the index of a nonzero component. The system was solved by the hybrid solver in a 3D rectangular domain *Ω* = [0,10]^3^ μm^3^, with the diffusion coefficient *D* = 1 μm^2^/s. Values of other parameters were correspond to Fig 2A in [53]: *r*_*c*_ = (0.3/(4π*L*_0_))^1/3^ ≈ 0.0341 μm; $a=b=\tau_{\text{D}}^{-1}$ with $\tau_{\text{D}}=r_{\text{c}}^{2}/D$ ≈ 1.163 × 10^−3^ s; *κ*_f_ = 4*πDr*_c_ ≈ 0.429 μm^3^ /s and $\kappa_{\text{r}}=\kappa_{\text{f}}L_{0}=0.3\tau_{\text{D}}^{-1}$ ≈ 258 s^-1^. As in [53], the macromolecules were initially unbound and equally partitioned between the inert and reactive states; thus *C*(0) = 0, and the relaxation function reduces to 1 − *C*(*t*)/*C*_eq_. The total number of macromolecules was *N* = 20000, equivalent to ≈ 3.322 × 10^−2^ μM, and the initial ligand concentration was *L*_0_ = 1 μM.

**Fig 7:**
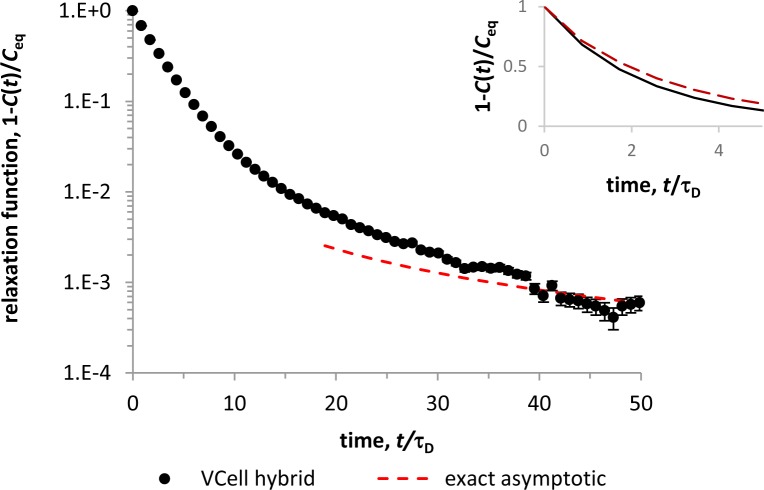
Hybrid solution of a system of stochastically-gated reactions, Eqs (6 and 7). For *t* >> τ_D,_ the probability that a macro-molecule remains unbound (dots with error bars) deviates from an exponential and approaches the power-law predicted in [53] (dashed curve). Inset: initial exponential decay of the relaxation function obtained with *h* = 0.2 μm (solid curve) and *h* = 0.034 μm (dashed curve).

The relaxation function, shown in Fig 7 as dots with error bars representing the standard deviation, was obtained from a hybrid solution based on 4052 realizations. The simulations were run with time step *Δt* = 10^−5^ s and mesh size *h* = 0.2 μm. In the simulations, the maximal depletion of ligands in the vicinity of particles was about 40%. The results of Fig 7 are similar to those presented in Fig 2A of [53]. The relaxation function starts as an exponential, but later approaches the predicted power-law asymptotic, $\frac{\kappa_{\text{f}}(1+a/b){\left(4\pi Dt\right)}^{-3/2}}{\kappa_{\text{r}}{\left(1+a/b+L_{0}\kappa_{\text{f}}/\kappa_{\text{r}}\right)}^{2}}$ (dashed curve in Fig 7). However, the solution significantly overestimates the steepness of the initial exponential decrease (solid curve in the inset of Fig 7), and the value of *C*_eq_ which was approximated as $C{|}_{t=110\cdot \tau_{\text{D}}}$. These readouts depend on accurate description of gradients of *L*(**r**,*t*) in the vicinity of the particles. Solving the model on a finer mesh with *h* = 0.034μm indeed improved the solution (dashed curve in the inset of Fig 7). In this case, we solved the problem in *Ω* = [0,1.7]^3^ μm^3^ with the same particle density, initial ligand concentration, and kinetic constants, and observed the maximal local depletion of ligands of about 75%. Still, the new solution differs noticeably from the result of [53], and decreasing *h* further is not necessarily helpful, since the mesh size is already close to *r*_*c*_. We therefore conclude that in this application, the accuracy of our hybrid method is largely limited by the fact that particles are approximated as points. Note also that the handling of strong depletion of continuous variables in the vicinity of discrete particles might be improved by employing multiscale approaches proposed for cases with spatial separation of subsystems with disparate levels of stochasticity [43–46]. Overall, the test shows that even at the limits of applicability, the method produces qualitatively, and in some respects quantitatively, reasonable results.

The VCell spatial hybrid solver also applies to models where continuous and discrete variables are defined both in the volume and in surrounding membranes. Subroutines supporting surface-bound stochastic sources were validated using a version of the model of calcium sparks, in which channels were placed on the cell membrane. For this case, the terms with stochastic variables move from the PDE to its boundary conditions. Diffusion on surfaces was rigorously validated separately in VCell [49] and Smoldyn.

### Application to a hybrid model of spontaneous cell polarization

In this section, we formulate a deterministic-stochastic model of spontaneous emergence of cell polarity and simulate it with our method. The model is a hybrid version of a fully stochastic mechanism originally proposed by Altschuler et al. [54].

Division, differentiation, and proliferation of living cells rely on mechanisms of symmetry breaking. A key element of these mechanisms is emergence of asymmetric (polar) distributions of signaling molecules, often in form of molecular clusters. While clustering may be spurred by external cues, many cell types can polarize spontaneously (see [54, 55] and references therein). Positive feedback in cell signaling is thought to play a crucial role in establishing cell polarity. The model by Altschuler et al. demonstrates that the positive feedback combined with stochasticity is sufficient for the emergence of a unipolar distribution of membrane-bound molecules. In the model, molecules from a cytoplasmic pool randomly associate with, and dissociate from, the membrane. While in the membrane, they diffuse but also recruit more molecules from the pool. The positive feedback reinforces the clustering. Remarkably, stochasticity of the system is critical for self-polarization: the effect disappears if the copy number of molecules in the membrane exceeds a certain threshold, so that there are no asymmetric solutions in the deterministic limit.

However, it is not uncommon for the membrane molecular clusters to involve large numbers of molecules. One such example is focal adhesions whose formation is initiated by membrane proteins called integrins. Activated by their binding to extracellular matrix, the integrins recruit many other molecules from the cytosol, which together form a focal adhesion. In our deterministic-stochastic model, the membrane receptor proteins that initiate clustering are distinguished from the cytosolic proteins recruited to the membrane. We assume that numbers of receptor proteins are sufficiently small to be represented by discrete variables, whereas copy numbers of cytosolic proteins, both recruited to the membrane and remaining in the cytoplasm, can be modeled continuously in terms of surface densities and volumetric concentrations. We then solve this hybrid model numerically using our method to determine if it retains the property of spontaneous polarization.

The corresponding ‘Langevin-like’ formulation of the problem is as follows. Consider a cell *Ω* with the plasma membrane ∂*Ω*. Let *U*(**r**,*t*) (**r** ∈ *Ω*) be the volume density of the proteins in the cytoplasm and *S*(**r**,*t*) (**r** ∈ ∂*Ω*) be the surface density of the proteins recruited to the membrane. To describe receptor proteins residing in the membrane, we introduce discrete variables *Γ*_*i*_(**r**,*t*) = δ(**r** − **r**_*i*_(*t*))*γ*_*i*_(*t*) with **r** ∈ ∂*Ω* and *i* = 1,…, *N*_*r*_, where *N*_*r*_ is the total number of receptors in the membrane. The discrete random variables *γ*_*i*_(*t*) accept two values: 0 (inactive receptor) and 1 (active receptor), whereas **r**_*i*_(*t*) are continuous random variables in ∂*Ω* (see discussion in subsection *capabilities and limitations of the method*).

Variables *U*(**r**,*t*) and *S*(**r**,*t*) form the ‘deterministic’ subsystem of the model and are governed by the following equations:

$$\begin{array}{l} \partial_{t}U=D_{U}\Delta U\\ \partial_{t}S=D_{S}\Delta_{s}S+k_{1}U\sum_{i=1}^{N_{r}}\Gamma_{i}-k_{2}S \end{array},$$ (8)

where *Δ* is the Laplacian in *Ω*, whereas *Δ*_*s*_ is the Laplace-Beltrami operator describing diffusion in ∂*Ω* (see, e.g., [49]); *D*_*U*_ and *D*_*S*_ are the corresponding diffusion constants. The two other terms in the equation for *S* are the rates with which the cytosolic proteins are recruited to, and dissociated from, the membrane; *k*_1_, *k*_2_ are the corresponding on- and off- rate constants. The boundary condition for the equation describing *U* reflects the local mass conservation,

$$-D_{U}(n\nabla U){|}_{\partial \Omega_{\text{cell}}}=-k_{1}U\sum_{i=1}^{N_{r}}\Gamma_{i}+k_{2}S,$$ (9)

where **n** is the outward normal.

Realizations of *γ*_*i*_(*t*) are governed by Poisson processes with the following transition probabilities:

$$\begin{array}{l} P(\Gamma_{i}{\left(r,t+dt\right)}_{\gamma_{i}=1}|\Gamma_{i}{\left(r,t\right)}_{\gamma_{i}=0})=k_{3}S(r,t)dt\\ P(\Gamma_{i}{\left(r,t+dt\right)}_{\gamma_{i}=1}|\Gamma_{i}{\left(r,t\right)}_{\gamma_{i}=1})=1-k_{4}dt\\ P(\Gamma_{i}{\left(r,t+dt\right)}_{\gamma_{i}=0}|\Gamma_{i}{\left(r,t\right)}_{\gamma_{i}=1})=k_{4}dt\\ P(\Gamma_{i}{\left(r,t+dt\right)}_{\gamma_{i}=0}|\Gamma_{i}{\left(r,t\right)}_{\gamma_{i}=0})=1-k_{3}S(r,t)dt \end{array},$$ (10)

where *k*_3_, *k*_4_ are the on- and off- rate constants for receptor activation. Stochastic variables **r**_*i*_(*t*) are modeled on an assumption that inactive receptors diffuse in the membrane, while active receptors are immobile. Accordingly,

$$r_{i}(t+dt)=\{\begin{array}{c} r_{i}(t)+dr(r_{i}(t),dt),\;\mathrm{if}\;\gamma_{i}(t)=0\\ r_{i}(t),\;\mathrm{if}\;\gamma_{i}(t)=1 \end{array},$$ (11)

where *d***r**(**r**_*i*_(*t*), *dt*) is a realization of a Wiener-type stochastic process described by Green’s function for the diffusion operator ∂_*t*_ − *D*_*Γ*_*Δ*_*s*_ on ∂*Ω*; the function is centered at **r**_*i*_(*t*). The initial positions of the receptors **r**_*i*_(0) are uniformly distributed in ∂*Ω*. Other initial conditions are discussed below.

The model includes a positive feedback between *Γ*_*i*_(**r**,*t*) and *S*(**r**,*t*), given that the rate of recruitment of cytosolic proteins to the membrane depends on *Γ*_*i*_(**r**,*t*), while the receptor activation rate depends on *S*(**r**,*t*). It is easy to see that the system described by Eqs (8–11) has an inactive steady state: *γ*_*i*_(*t*) = 0 for all *i*, *S*(**r**,*t*) = 0, and *U*(**r**,*t*) = *U*_0_ (*U*_0_ is the initial uniform concentration of the cytosolic protein). For some parameter sets, however, the inactive steady state can become unstable or the model may exhibit multi-stability. These possibilities can be explored by solving the model with varying initial conditions. Alternatively, one can transiently perturb the inactive steady state used as an initial condition. The latter approach was implemented in the example below by adding a pre-activation pulse to the intrinsic activation rate $P(\Gamma_{i}{\left(r,t+dt\right)}_{\gamma_{i}=1}|\Gamma_{i}{\left(r,t\right)}_{\gamma_{i}=0})=(k_{0}e^{-t/\tau }+k_{3}S(r,t))dt$ and, correspondingly, $P(\Gamma_{i}{\left(r,t+dt\right)}_{\gamma_{i}=0}|\Gamma_{i}{\left(r,t\right)}_{\gamma_{i}=0})=1-(k_{0}e^{-t/\tau }+k_{3}S(r,t))dt$; *k*_0_ and *τ* are the rate and time constants of the pulse.

**Fig 8:**
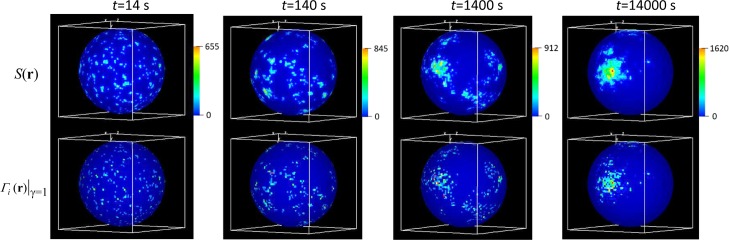
Cell polarization: coalescence of a multi-cluster system into a single cluster. Distributions of proteins recruited to the membrane from the interior (top) and active receptors (bottom), obtained by solving the model of Eqs (8–11) with VCell spatial hybrid for parameters specified in the text. Local surface densities increase in large clusters, as the number of clusters diminishes (see color scales).

The model has been solved by the spatial hybrid method in a spherical cell with radius *R* = 4 μm for the following model parameters: *U*_0_ = 1 μM, *N*_*r*_ = 1000, *D*_*U*_ = 10 μm^2^/s, *D*_*S*_ = *D*_*Γ*_ = 0.1 μm^2^/s, *k*_1_ = 0.01 μM^-1^s^-1^, *k*_2_ = 0.01 s^-1^, *k*_3_ = 0.01 μm^-2^s^-1^, *k*_4_ = 0.1 s^-1^. For this parameter set, the inactive state is unstable: activation of a single receptor drives the system to its active state with an average of about 800 active receptors. Interestingly, spatial averages of all variables have reached their active steady-state regimes relatively quickly (by *t* = 10 s, for the robust pre-activation characterized by *k*_0_ = 10 s^-1^ and *τ* = 1 s, and by *t* ≈ 350 s, when just ten receptors were initially activated), whereas the cluster structure evolves on a much longer time scale, see results in Fig 8 obtained for *k*_0_ = 10 s^-1^ and *τ* = 1 s.

As in the original stochastic model [54], the hybrid mechanism yields a spatially heterogeneous steady state with a single cluster of activated receptors and recruited proteins. But unlike the original model, the total number of proteins in clusters can be large, because the condition of small copy numbers applies in the hybrid model only to the receptors initiating the clustering. Note the increase of local densities in the surviving clusters (see color scales in Fig 8), which is consistent with the early stabilization of spatial averages. While the ‘attractive’ spatial correlations of active receptors originate from the positive feedback, a corresponding deterministic formulation does not yield a spatially heterogeneous steady state (as was the case with the original model [54]), indicating that the discreteness and stochasticity of the receptors also play an essential role in establishing the polar distributions of membrane-bound molecules. Interestingly, the kinetics of cell polarization predicted by the model is reminiscent of glassy behavior, in which a system approaches a stable steady state by going through a long sequence of metastable states [56].

## Methods

The deterministic-stochastic algorithm described in this article integrates a spatial particle-based fixed time step Monte Carlo method (Smoldyn) and a conventional PDE solver with compatible time-stepping (one of the VCell solvers). The PDE solver utilizes finite-volume spatial discretization of PDEs [48, 49], which ensures local mass conservation, and a semi-implicit time discretization scheme, in which the diffusion/ advection operator applies to variables at time *t* + *Δt* while the reaction and membrane flux terms are evaluated at time *t* [50, 51]. To ensure consistency in handling geometry by the two methods, triangulation of surfaces is performed by applying Taubin smoothing [57] to watertight pixilated surfaces emerging from segmentation of space. The approach is applicable both to geometries defined analytically and to irregular realistic geometries based on experimental images.

Implementation in VCell Math workspace of the hybrid model of spontaneous cell polarization described in Section *Application to a hybrid model of spontaneous cell polarization* is detailed in S3 Text. The corresponding VCell MathModel, ‘Hybrid_cell_polarity_public’, along with simulation results, can be found by logging to VCell, http://vcell.org, and searching the database of public MathModels under username ‘boris’.

## Discussion

Stochastic processes are ubiquitous in cellular systems. A deterministic-stochastic description of interacting components with disparate degrees of stochasticity provides an efficient alternative to a full stochastic treatment of the problem. In a hybrid numerical approach, an appropriate integration of deterministic and stochastic methods yields significant computational savings.

In this paper, we describe a general-purpose hybrid method for solving spatial deterministic-stochastic models in realistic cell geometries. The emphasis is placed on the physical fundamentals of the method and its testing. The method is based on a formulation in terms of stochastic variables of two types: continuous variables, described by partial differential equations with stochastic source terms, and discrete variables governed by stochastic jump processes. Numerically, the algorithm is a Monte Carlo fixed time step integrator generating realizations of the hybrid system. The current implementation utilizes a VCell fixed time step PDE solver coupled with a particle-based stochastic simulator Smoldyn.

Validating a hybrid deterministic-stochastic numerical scheme is conceptually nontrivial and logistically challenging. We tested our method against analytical results and numerical solutions obtained by alternative methods. The expected convergence of solution error was observed in tests with a separable stochastic subsystem. Testing of the method in conditions of full coupling was performed in the limit of fast diffusion against well-mixed solutions obtained with nonspatial Gibson-Bruck method and against a direct solution of a corresponding Fokker-Planck equation. The latter approach was also used for testing spatially heterogeneous solutions of fully coupled hybrid systems.

The method has been applied to a hybrid model of spontaneous cell polarization based on the original idea of Altschuler et al. [54]. The solution recapitulates emergence of a stable asymmetric distribution of membrane-bound molecules, as a result of positive feedback and stochasticity. But in the hybrid version, the total number of membrane molecules is free from the small copy number requirement, which now applies only to the number of receptors that initiate clusters. The model predicts glassy-like kinetics of coalescence of the multi-cluster structure into a single cluster.

While the VCell spatial hybrid solver is practical for many typical applications, its performance may become suboptimal for cases with disparate time scales (‘stiff’ problems), as the integration is done with a fixed time step. The handling of the discrete variables can be optimized by incorporating adaptive approaches, although potential savings should be weighed against costs associated with additional logistical complexity, particularly since the inefficiencies are often caused by stiffness of the deterministic subsystem. While the stiffness caused by fast reactions that persist throughout the time of interest can be addressed by applying the VCell automatic quasi-steady-state approximation (see discussion in Section *Mathematical problem and algorithm*), the treatment of continuous variables would generally benefit from implementation of time-step control commonly employed in deterministic numerical algorithms.

## Supporting Information

S1 Text Exact mathematical expectation value of the spatial average of the ‘deterministic variable in a hybrid model with separable subsystems (DOCX)

S2 Text Discretization of Eq (5) and solution of discretized equations (DOCX)

S3 Text Implementation of the hybrid model of spontaneous cell polarization in VCell Math Workspace (DOCX)
