# Supplementary material for: Numerical Approach to Spatial Deterministic-Stochastic Models Arising in Cell Biology
Source: PLoS Comput Biol. 2016 Dec 13;12(12):e1005236. doi: 10.1371/journal.pcbi.1005236 (PMC5154471; doi:10.1371/journal.pcbi.1005236)
Supplement: S1 Text — (DOCX) [file pcbi.1005236.s001.docx]

**Text S1. Exact mathematical expectation value of the spatial average of the ‘deterministic variable in a hybrid model with separable subsystems**

For the case of a separable stochastic subsystem of channels with parameters$k_{\text{on}}^{i}$and $k_{\text{off}}^{i}$ that are independent of $U(\mathbf{r},t)$, integration of Eqs (1-3) of the main text yields for $\overline{U}_{\text{exact}}(t)=$ $E[|\Omega_{\text{cell}}|^{-1}\int_{\Omega_{\text{cell}}} U(\mathbf{r},t)d\mathbf{r}]$,

$\bar{U}_{\text{exact}}(t)=U_{0}+\frac{N_{\text{ch}}Jk_{\text{on}}}{|\Omega_{\text{cell}}|(k_{\text{on}}+k_{\text{off}})}\left\{\frac{1-\exp(-V_{\text{p}}t)}{V_{\text{p}}}+\frac{\exp(-(k_{\text{on}}+k_{\text{off}})t)-\exp(-V_{\text{p}}t)}{k_{\text{on}}+k_{\text{off}}-V_{\text{p}}}\right\}$,

where $|\Omega_{\text{cell}}|$ is the cell volume.
