# Supplementary material for: Numerical Approach to Spatial Deterministic-Stochastic Models Arising in Cell Biology
Source: PLoS Comput Biol. 2016 Dec 13;12(12):e1005236. doi: 10.1371/journal.pcbi.1005236 (PMC5154471; doi:10.1371/journal.pcbi.1005236)
Supplement: S2 Text — (DOCX) [file pcbi.1005236.s002.docx]

**Text S2. Discretization of Eqs (5) and solution of discretized equations**

Given the increments $\Delta x$ and $\Delta\rho$, the problem domain is discretized by first meshing the geometry, $x_{i}=i\Delta x$, $i=0,\text{ }1,\text{ }...,\text{ }i_{\max}-1$, and then discretizing the ‘mass’ variables $m^{(i)}=\rho(x_{i})\Delta x$: $m_{n}^{(i)}=n_{i}\Delta\rho\Delta x$, $n_{i}=0,\text{ }1,\text{ }...,\text{ }n_{\max}(i)-1$. The resulting points $\{n_{i}\}=$ $(\{n_{0}\},\{n_{1}\},...,\{n_{i_{\max}-1}\})$form an $i_{\max}$-dimensional grid. A discrete version of Eqs (5) is formulated on this grid in terms of the probabilities $P_{k}(\{n_{i}\},\tau)=$ $p_{k}(\{n_{i}\Delta\rho\},\tau)\Delta\rho^{i_{\max}}$governed by the following equations,

$\begin{matrix} \partial_{\tau}P_{k}=(-1)^{k}\alpha(P_{1}-\beta(n_{0}\Delta\rho+1)P_{0})+\frac{a}{\Delta\rho\Delta x}\text{δ}_{k,1}(P_{1}(n_{0}-1,...)-P_{1})+ \\ \sum_{i} [D(\Delta x)^{-2}((n_{i}+1)(P_{k}(...,n_{i}+1,n_{i+1}-1,...)+P_{k}(...,n_{i-1}-1,n_{i}+1,...))-2n_{i}P_{k})+ \\ (n_{i}+1))P_{k}(...,n_{i}+1,...)-n_{i}P_{k}],\text{ }k=0,\text{ }1 \end{matrix}$ (T2.1)

where $\text{δ}_{k,1}$ is the Kronecker symbol and $P_{k}\equiv P_{k}(\{n_{i}\},\tau)$. Note that the discretization of the diffusion operator in Eqs (T2.1), while formally similar to that of the reaction-diffusion master equation, applies here to a ‘deterministic’ variable. Eqs (T2.1) were solved with a custom MATLAB code optimized for handling large sparse matrices. Accuracy of the solutions of Eqs (T2.1) was limited by feasible sizes of the grid.

The solution of Eqs (T2.1), shown as solid curves in Figure 5, was obtained for $[n_{\max}(0),n_{\max}(1)]=$ [1600, 200], with$\Delta\rho=$1.25e-3 and $\Delta\tau=$3.1e-4.

The extrapolated curves shown in Figure 6 are based on numerical solutions of the Fokker-Planck equation obtained for six three-dimensional grids $[n_{\max,s}(0),n_{\max,s}(1),_{\max,s}(2)]$= [80,72,65], [98,83,74], [120,108,97], [147,119,106], [180,146,130], and [221,179,159], with the corresponding $\Delta\rho_{s}=\rho_{\max}(0)/n_{\max,s}(0)$ and $\Delta\tau_{s}=$ $0.0025\Delta\rho_{s}$, where $\rho_{\max}(0)=$ 2.55 and $s=$0,1,..5 (note that $\Delta\rho_{s}$and $\Delta\tau_{s}$ varied approximately as $\propto(1.5)^{-s}$). The extrapolated values were determined for each $n_{i}$by fitting the values of the marginal probability densities $y_{s}(n_{i})\equiv p_{s}(n_{i},\tau)$($s=$0,1,..5) to a second-degree polynomial. The fitting yielded, in agreement with the discretization in Eqs (T2.1), the nearly linear polynomials, whose constant terms are the sought extrapolated values.
