# Supplementary material for: Numerical Approach to Spatial Deterministic-Stochastic Models Arising in Cell Biology
Source: PLoS Comput Biol. 2016 Dec 13;12(12):e1005236. doi: 10.1371/journal.pcbi.1005236 (PMC5154471; doi:10.1371/journal.pcbi.1005236)
Supplement: S3 Text — (DOCX) [file pcbi.1005236.s003.docx]

**Text S3. Implementation of the hybrid model of spontaneous cell polarization in VCell Math Workspace**

MathDescription {

Constant D 10.0;

Constant D1 0.1;

Constant D2 0.0;

Constant D3 0.1;

Constant k0 10.0;

Constant k1 0.01;

Constant k2 0.01;

Constant k3 0.01;

Constant k4 0.1;

Constant N 1000.0;

Constant tau 1.0;

Constant U_init 1.0;

Constant S_init 0.0;

VolumeVariable cell::U

MembraneVariable cell_extra_membrane::S

MembraneParticleVariable cell_extra_membrane::Gc

MembraneParticleVariable cell_extra_membrane::Go

Function flux ((Go * k1 * U) - (k2 * S));

CompartmentSubDomain cell {

BoundaryXm Flux

BoundaryXp Flux

BoundaryYm Flux

BoundaryYp Flux

BoundaryZm Flux

BoundaryZp Flux

PdeEquation U {

Rate 0.0;

Diffusion D;

Initial U_init;

}

}

CompartmentSubDomain extra {

BoundaryXm Flux

BoundaryXp Flux

BoundaryYm Flux

BoundaryYp Flux

BoundaryZm Flux

BoundaryZp Flux

}

MembraneSubDomain cell extra {

BoundaryXm Value

BoundaryXp Value

BoundaryYm Value

BoundaryYp Value

BoundaryZm Value

BoundaryZp Value

PdeEquation S {

Rate (602.0 * flux);

Diffusion D3;

Initial S_init;

}

ParticleProperties Gc {

ParticleInitialCount {

ParticleCount N;

ParticleLocationX u;

ParticleLocationY u;

ParticleLocationZ u;

}

ParticleDiffusion D1;

}

ParticleProperties Go {

ParticleInitialCount {

ParticleCount 0.0;

ParticleLocationX u;

ParticleLocationY u;

ParticleLocationZ u;

}

ParticleDiffusion D2;

}

ParticleJumpProcess open {

SelectedParticle Gc

MacroscopicRateConstant ((k0 * exp ((- t / tau))) + (k3 * S));

Effect Gc DestroyParticle

Effect Go CreateParticle

}

ParticleJumpProcess close {

SelectedParticle Go

MacroscopicRateConstant k2;

Effect Go DestroyParticle

Effect Gc CreateParticle

}

JumpCondition U {

InFlux - flux;

OutFlux 0.0;

}

}

}

Model geometry is introduced separately through a user interface:

Domain: 3D, size= (8.4, 8.4, 8.4), origin= (0.0, 0.0, 0.0)

Subdomains: “cell” ((x – 4.2) ^ 2.0 + (y – 4.2) ^ 2.0 + (z – 4.2) ^ 2.0) < (4.0 ^ 2.0);

“extra” 1.0.
